# Biocompatibility of a New Calcium Silicate-Based Root Canal Sealer Mediated via the Modulation of Macrophage Polarization in a Rat Model

**DOI:** 10.3390/ma15051962

**Published:** 2022-03-07

**Authors:** Xiaoqian Yang, Jun Tian, Mengjie Li, Weiyang Chen, He Liu, Zhejun Wang, Markus Haapasalo, Ya Shen, Xi Wei

**Affiliations:** 1 Hospital of Stomatology, Guanghua School of Stomatology, Sun Yat-Sen University, Guangzhou 510055, China; yangxq6@mail2.sysu.edu.cn (X.Y.); tianjun2@mail2.sysu.edu.cn (J.T.); lmengj@mail2.sysu.edu.cn (M.L.); chenwy27@mail2.sysu.edu.cn (W.C.); 2 Guangdong Provincial Key Laboratory of Stomatology, Guangzhou 510080, China; 3 Department of Stomatology, Affiliated Hospital of Jining Medical University, Jining 272000, China; endoliuhe@gmail.com; 4 Division of Endodontics, Department of Oral Biological & Medical Sciences, Faculty of Dentistry, The University of British Columbia, Vancouver, BC V6T 1Z3, Canada; zhejun@dentistry.ubc.ca (Z.W.); markush@dentistry.ubc.ca (M.H.)

**Keywords:** animal model, biocompatibility, EndoSequence BC Sealer HiFlow, inflammation, macrophage polarization, subcutaneous connective tissue

## Abstract

(1) Background: The EndoSequence BC Sealer HiFlow (Brasseler, Savannah, GA, USA) has recently been introduced in clinical applications. Thus, the aims of the present study are to determine its biocompatibility in vivo and to examine its ability to drive macrophage polarization in vitro and in vivo. (2) Methods: HiFlow was implanted into rat connective tissue for 7, 30 and 150 days. The microstructures and elemental compositions were determined by scanning electron microscopy-energy-dispersive X-ray spectroscopy (SEM-EDX). Hematoxylin–eosin, immunofluorescence, RT–qPCR and flow cytometry were used to elucidate the effects on inflammatory responses and macrophage polarization. (3) Results: SEM-EDX revealed the formation of surface hydroxyapatite crystal layers. Histological evaluation showed that HiFlow exhibited long-term biocompatibility because it decreased inflammatory responses and reduced the number of macrophages over time; however, tissue necrosis was observed in all the groups. RT–qPCR verified that HiFlow regulated the expression of inflammatory factors to inhibit the inflammatory response. Immunofluorescence analysis performed on in vivo samples revealed that HiFlow promoted M2-like macrophage polarization, and these results were confirmed by flow cytometry in vitro. (4) Conclusion: After 150 days of investigation, HiFlow was considered biologically acceptable, and the formation of apatite crystal layers and the promotion of M2-like macrophage polarization may contribute to its favorable biocompatibility.

## 1. Introduction

Root filling sealers that are applied during root canal treatment should have good biocompatibility and sealing ability and exert good osteoinductive effects to promote the healing of apical periodontitis. The calcium silicate-based bioceramic root canal sealer iRoot SP (Innovative BioCeramix Inc., Vancouver, BC, Canada), also called Endosequence BC Sealer (Brasseler, Savannah, GA, USA), was introduced to the field of endodontics, and it has attracted considerable attention due to its ease of use. This material is a premixed, injectable, and ready-to-use white cement paste that is mainly composed of calcium silicate, calcium phosphate, calcium hydroxide, zirconium oxide, filler, and thickening agents [1]. This sealer exhibits favorable flowability, excellent sealing abilities, and good biocompatibility, and it exerts good osteoconductive effects [2,3,4,5]. Furthermore, iRoot SP is hydrophilic and requires liquid from dentinal tubules or periapical tissues to set and harden without shrinking [3,6]. Thus, the manufacturer recommends using the single-cone (SC) technique, which has been suggested to be a viable option for root canal obturation.

However, whether iRoot SP is suitable for use with continuous wave of condensation (CWC) or warm vertical compaction techniques is currently controversial. It has been reported that the use of iRoot SP with the CWC technique is more effective than its use with the SC technique for artificially filling lateral canals [7]. However, the CWC technique decreases the bond strength of iRoot SP [8]. Moreover, the high temperatures used during warm vertical compaction result in dramatic reductions in the setting time and flowability of bioceramic-based sealers, including iRoot SP, most likely negatively affecting the quality of obturation [9].

To overcome the limitation of high temperature, the EndoSequence BC Sealer HiFlow (Brasseler, Savannah, GA, U.S.A.), a novel high temperature-resistant bioceramic root-filling paste, was recently introduced. The manufacturer claims that HiFlow exhibits better flowability and lower viscosity when heated to 150–220 °C and that it is more radiopaque, making it an optimal choice for the warm vertical obturation technique. Our previous study demonstrated that HiFlow combined with the CWC technique resulted in a significantly higher percentage of sealer penetration area than iRoot SP combined with the SC technique at a distance of 4 mm from the apex, and HiFlow combined with the CWC technique penetrated deeper into dentinal tubules than iRoot SP combined with the SC technique at both the 8 mm and 12 mm levels [10]. Moreover, another study reported that the percentage of void volumes and root canal gaps in the HiFlow/CWC group was lower than that in the HiFlow/SC group. Thus, the combined use of HiFlow with the CWC technique may be a worthwhile choice for root canal treatment [11]. In vitro experiments have recently been carried out by many scholars to confirm the biocompatibility of HiFlow [12,13]. The long-term in vivo biocompatibility and toxicity of HiFlow should also be thoroughly studied before it is widely used in the clinic. Many previous studies have been performed to evaluate the in vivo biocompatibility of root canal sealers in a rat model of subcutaneous tissue implantation [4,14]. Therefore, the aims of the present study are to evaluate the reactions of subcutaneous connective tissues and organs to BC Sealer HiFlow and to compare them to the reactions to iRoot SP sealer and ProRoot mineral trioxide aggregate cement (MTA) (Dentsply, Tulsa, OK, USA), whose sufficient biocompatibilities have been confirmed by numerous in vivo and in vitro experiments [15,16]. Additionally, macrophages, the main cells that infiltrate the interface between implanted materials and tissues, secrete proinflammatory and anti-inflammatory cytokines to regulate the foreign body reaction, and we also studied the effects of iRoot SP and BC Sealer HiFlow on macrophage polarization [17]. The null hypotheses tested were as follows: 1) the new calcium silicate-based root canal sealer BC Sealer HiFlow does not exhibit biocompatibility; and 2) there are no differences in the abilities of HiFlow and the other calcium silicate materials to drive macrophage polarization.

## 2. Materials and Methods

The procedure for tooth extraction was approved by the Ethics Committee for Research with Human Beings of Guanghua School of Stomatology (Guangzhou, China) (No. KQEC-2020-62-01), and the same research protocol was approved by the Animal Ethics Screening Committee of Sun Yat-sen University in Guangzhou, China (No. SYSU-IACUC-2020-000090). Twenty-four young adult male Sprague–Dawley (SD) rats, aged 2–4 months and weighing 180–250 g, were randomly used at each time point for the biocompatibility animal experiment (*n* = 8 per period) based upon methods described in previous studies [4,18]. A qualified veterinarian was responsible for caring for and feeding these rats in the Laboratory Animal Center.

### 2.1. Preparation of Specimens

A total of 96 permanent single-rooted human teeth with straight roots were extracted and stored in a saline solution in an ice bath for 10 min, while in transit to the laboratory. The crowns of all the teeth were removed, and one-third of each root was shortened to 2 mm with an inner diameter of 2 mm using the same bur. The dentine tubes were then cleaned with 3% NaClO for 5 min and washed with sterile distilled water before being autoclaved. Then, dentine tubes filled with HiFlow (Brasseler, Savannah, GA, USA), iRoot SP (Innovative BioCeramix Inc., Vancouver, BC, Canada), or ProRoot MTA (Dentsply, Tulsa, OK, USA) or empty dentine tubes (control) were incubated for 24 h at 37 °C in a humidified chamber with 5% CO_2_ to allow semisolidification. Then, the dentine tubes were implanted into 24 rats. ProRoot MTA was mixed manually, while the two sealers were automatically injected into the 2 mm wide canals in the root dentine tubes.

### 2.2. Tube Implantation

After the rats were anaesthetized by intraperitoneal injection of 1% sodium pentobarbital (40 mg/kg body weight), the dorsal skins were shaved and disinfected using an alcohol–iodine solution. Four blind pouches were formed along both sides of the dorsal spine, and these pouches were prepared by blunt dissection with 1 cm incisions. Dentine tubes were filled with each endodontic material and incubated at 37 °C in 100% relative humidity for 24 h. Then, the specimens were inserted into three different pouches at a certain distance of more than 1 cm from the incision. The negative control was the implantation of an empty dentine tube into the fourth pouch. The incisions were closed with absorbable sutures, and the animals were housed in a specific pathogen-free (SPF) environment and given access to a regular diet and drinking water until the experiments were performed. Once every two days, the condition of the healing wounds was routinely observed, and food, water and padding were replaced in order to provide the best care to the animals and obtain adequate experimental data.

### 2.3. Analysis of Dentine Tube Sealer Composition and Structure

One hundred fifty days after implantation, all the animals were euthanized by an overdose of anesthesia. The tube specimens were removed, and their surfaces were sputtered with gold and analyzed by scanning electron microscopy-energy-dispersive X-ray spectrometry (SEM-EDX) (Gemini 500; ZEISS, Oberkochen, Baden-Württemberg, Germany).

### 2.4. Analysis of Systemic Toxicity

After 30 and 150 days, the organs, including the liver, spleen, kidney, lung and heart, were harvested and stained with hematoxylin–eosin (H&E) to evaluate toxicity via optical microscopy observation (Axiostar plus; Carl Zeiss Meditec AG, Jena, Germany) at different magnifications (100× and 400×). Normal untreated rats served as the negative control group.

### 2.5. Analysis of Inflammatory Responses and Foreign Body Reactions

After 7, 30 and 150 days of investigation, the animals (*n* = 8 per time point) were euthanized by an overdose of anesthesia. The epidermis and subcutaneous connective tissues of each rat were resected from sites within a 1 cm diameter of the implanted dentine tubes and fixed in 4% paraformaldehyde for 24 h. Next, the tubes were removed and immersed in 2.5% glutaraldehyde. The tissue samples were cut in half, and the tissue halves were dehydrated, removed, impregnated and embedded in paraffin. Serial 4 μm thick sections were cut and stained with H&E to observe inflammatory cell infiltration.

Sections were analyzed at different magnifications (100× and 400×) by two calibrated evaluators who were blinded to the treatments using a slide scanner (Aperio AT2; Leica Biosystems, Vista, CA, USA). Histological assessment was performed on all samples, and the most central section received the most attention. Inflammatory infiltration was scored according to the following scale: (0) no inflammatory cells or no more than 10 inflammatory cells/area; (1) mild inflammation with the number of inflammatory cells ranging from 11 to 25 cells/area; (2) moderate inflammation with 26 to 65 inflammatory cells/area; and (3) severe inflammation with more than 65 inflammatory cells/area [4].

The same analytical method was used for the classification of foreign body reactions: (0) no macrophages or no more than 10 macrophages/area; (1) slight foreign body reaction with 10 to 30 macrophages/area; and (2) severe foreign body reaction with more than 30 macrophages/area. Necrotic areas were classified as necrosis being either (0) absent or (1) present [18].

### 2.6. Analysis of Macrophage Polarization In Vivo

To prepare fresh-frozen tissue sections, the other half of the tissue samples mentioned above were dehydrated in a gradient of sucrose solutions and embedded in optimal cutting temperature (OCT) compound (Sakura Finetek, Torrance, CA, USA) after fixation with 4% paraformaldehyde overnight at 4 °C. Serial 5 μm thick sections were cut and air dried for 30 min, washed with PBS and permeabilized with 0.1% Triton X-100. After blocking with serum-based blocking buffer for 60 min, the slides were incubated with primary antibodies against CD86 (Novus Biologicals, Centennial, CO, USA) and CD163 (Novus Biologicals, Littleton, CO, USA) overnight. After separately incubating the slides with Alexa Fluor 568-conjugated and Alexa Fluor 488-conjugated secondary antibodies (1:1000; Invitrogen, Waltham, MA, USA) for 60 min at room temperature, the slides were mounted with FluoroShield mounting medium containing 4′,6-diamidino-2-phenylindole (Abcam, Cambridge, UK). CD86-positive M1 macrophages and CD163-positive M2 macrophages were visualized with an Olympus FV3000 confocal microscope at a magnification of 400×, and the numbers of these macrophage populations were quantified using ImageJ software (ImageJ 1.51v, National Institutes of Health, Bethesda, MD, USA).

### 2.7. In Vitro Macrophage Polarization Analysis

#### 2.7.1. Isolation of Bone-Marrow-Derived Macrophages (BMDMs)

To isolate and culture BMDMs, marrow-derived nucleated cells were flushed from the femurs and tibias of C57BL/6J mice (between 6 and 12 weeks of age) and then grown in RPMI-1640 medium supplemented with 10% fetal bovine serum (FBS) and 20% L929 supernatant. Nonadherent cells were removed after 48 h, and the attached cells were maintained for an additional 5 days.

#### 2.7.2. RT–qPCR Assessment of the Effects of iRoot SP and HiFlow Extracts on the Expression of Inflammatory Mediators

The iRoot SP and BC Sealer HiFlow were placed into separate culture dishes and flattened to produce very thin discs. The specimens were allowed to solidify in a humidified atmosphere of 5% CO_2_/95% air for 48 h at 37 °C. Each disc was then crushed into a very fine powder using an agate mortar and pestle. To prepare hydrated iRoot SP and BC Sealer HiFlow extracts, 1 g of each powder was eluted in 50 mL of α-minimum essential medium (α-MEM, Invitrogen) at 37 °C for 24 h. Differentiated BMDMs were stimulated with 50% diluted bioceramic extracts in the presence or absence of lipopolysaccharide (LPS) for 12 h. An RNA-Quick Purification Kit (Yishan, Baoshan, Shanghai, China) was used to extract total RNA according to the manufacturer’s instructions. cDNA was then synthesized using the PrimeScriptTM RT reagent Kit (TaKaRa Co., Kyoto, Japan). Real-time PCR was performed using Fast SYBR Green Master Mix (Thermo Fisher, Waltham, MA, USA) and gene-specific primers. The results were obtained as threshold cycle values (Ct). Then, the mean Ct values of two independent measurements were used to calculate the relative mRNA expression levels by the 2^−∆∆CT^ method. The primer sequences are shown in Table 1, and the relative expression levels of mRNA were normalized to that of *β-actin*.

#### 2.7.3. Analysis of BMDM Surface Marker Expression by Flow Cytometry

After stimulation with bioceramic extracts for 12 h, BMDMs were washed with PBS (Gibco, Invitrogen, Paisley, UK), treated with TrypLE (Gibco) and resuspended in 0.5 mL of PBS supplemented with 3% FBS. Macrophage surface marker expression was analyzed using a flow cytometry system (BD Bioscience, San Jose, CA, USA) after staining with antibodies specific for CD86, CD163 and CD206 (PE-conjugated anti-mouse antibodies, eBioscience, San Diego, CA, USA) for 30 min in the dark at 4 °C.

### 2.8. Statistical Analysis

All cells were counted visually by two experienced evaluators who were blinded to the groupings, and the data are expressed as the mean ± SD. The results of H&E staining were statistically analyzed by Fisher’s exact and Kruskal–Wallis tests, while semiquantitative analysis was performed for immunofluorescence staining. The significance level was set to *p* ˂ 0.05 using SPSS software (version 26.00; SPSS Science, Chicago, IL, USA). A kappa test was performed by two evaluators who were blinded to the treatments.

## 3. Results

All the animals maintained a good general health status throughout the experimental period, and no significant differences in mean body weight were observed among all the groups before they were euthanized by an overdose of anesthesia (data not shown).

### 3.1. Apatite Layers Formed on the Surfaces of MTA, iRoot SP and HiFlow In Vivo

**Figure 1:**
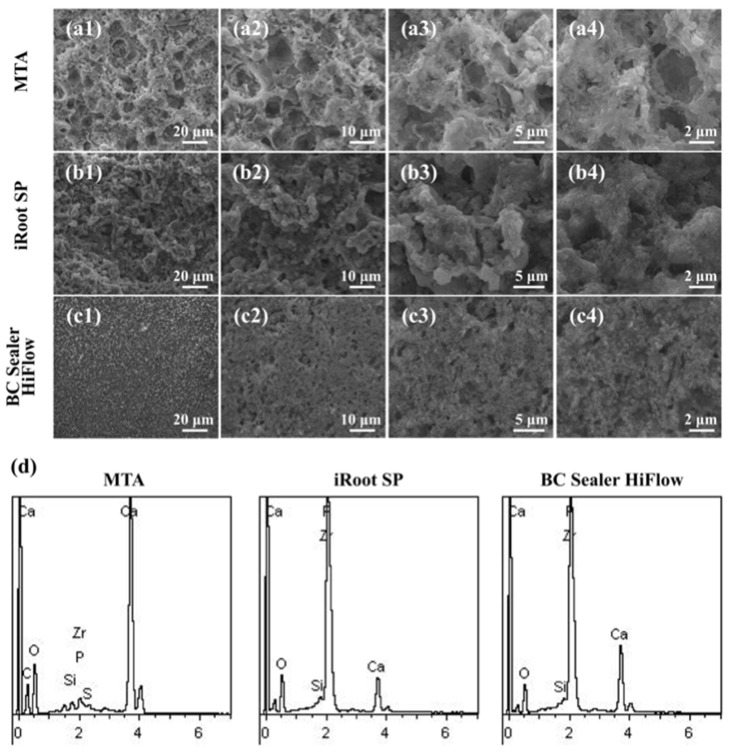
Analysis of in vivo surface characteristics of dentine tube biomaterials. SEM micrographs of ProRoot MTA (**a1**–**a4**); iRoot SP (**b1**–**b4**); EndoSequence BC Sealer HiFlow (**c1**–**c4**) at 4 magnifications (1000×, 2000×, 5000×, and 10,000×). (**d**) EDX spectrum of ProRoot MTA, iRoot SP and BC Sealer HiFlow.

The formation of hydroxyapatite crystals on the surface of biomaterials is vitally important for their biocompatibility and bioactivity. Therefore, SEM was used to observe the superficial ultrastructure, and energy spectrum analysis was used to determine the elemental composition 150 days after subcutaneous transplantation. SEM analysis (Figure 1) revealed that, after implanting the materials below the subcutaneous connective tissue, similar clumpy and cloudy particles were deposited on the surfaces of MTA, iRoot SP and BC Sealer HiFlow; among these materials, BC Sealer HiFlow exhibited the tightest arrangement, and the MTA surface was less dense and more porous than the surfaces of the other materials. The formation of apatite layers on these surfaces indicated the excellent biocompatibility of all the materials in vivo [19]. Moreover, EDX analysis (Table 2) of the precipitates revealed that all the samples displayed strong peaks for oxygen (O) and calcium (Ca) and a weak peak for silicon (Si). Additionally, a weak peak was observed for phosphorus (P) in the MTA and iRoot SP groups, while the HiFlow group had a strong peak for P. In addition, MTA contained larger amounts of carbon and calcium, but not of zirconium.

### 3.2. MTA, iRoot SP and HiFlow Exhibited No Systemic Toxicity

**Figure 2:**
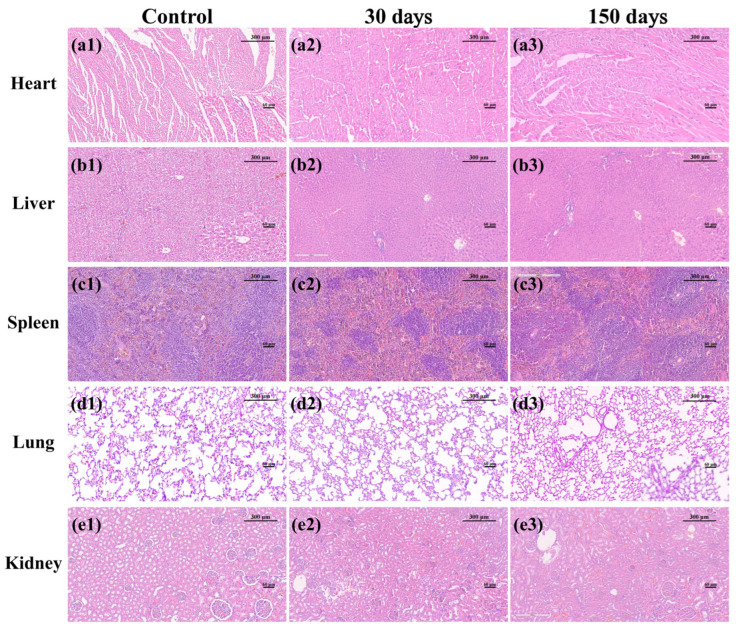
In vivo systemic toxicity associated with three biomaterials. (**a1**–**a3**) H&E staining of the hearts in the control, 30-day and 150-day groups. (**b1**–**b3**) H&E staining of the livers in the control, 30-day and 150-day groups. (**c1**–**c3**) H&E staining of the spleens in the control, 30-day and 150-day groups. (**d1**–**d3**) H&E staining of the lungs in the control, 30-day and 150-day groups. (**e1**–**e3**) H&E staining of the kidneys in the control, 30-day and 150-day groups. No significant inflammatory infiltration or tissue necrosis was observed. *p* ˃ 0.05 versus the normal group. (100×, bar, 300 μm; insets show tissue details at 400×, bar, 60 μm).

After investigating the biomaterial surface characteristics to obtain a preliminary understanding of their possible effects, it was logical to study their systemic and local toxicity. The systemic toxicity results are shown in Figure 2. Mild focal inflammatory cell infiltration was observed in the heart, liver, spleen, lung and kidney in the control group. Notably, focal inflammatory cell infiltration is commonly seen in the major organs of healthy animals. The exposure to MTA, iRoot SP and BC Sealer HiFlow for 30 and 150 days caused no significant physiological changes in the heart, liver, spleen, lung or kidney compared to exposure to control conditions, and no visible necrotic areas were observed, illustrating that no significant systemic toxicity was caused by any of the tested materials.

### 3.3. Long-Term Biocompatibility of MTA, iRoot SP and HiFlow

**Figure 3:**
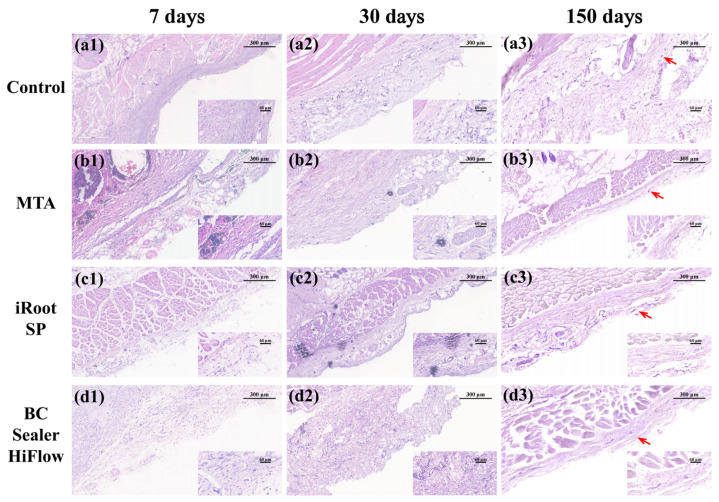
In vivo tissue response to three biomaterials. (**a1**–**d1**) H&E staining of subcutaneous connective tissues in the control, MTA, iRoot SP and BC Sealer HiFlow groups at 7 days. All the groups exhibited mostly moderate-to-severe inflammation. MTA exposure resulted in more visual fields with severe inflammation, whereas iRoot SP followed and BC Sealer HiFlow resulted in the lowest number of fields with severe inflammation. (**a2**–**d2**) H&E staining of subcutaneous connective tissues in the control, MTA, iRoot SP and BC Sealer HiFlow groups at 30 days. The control and MTA treatments resulted in mild inflammation. The SP and HiFlow treatments resulted in mostly mild-to-moderate inflammation. (**a3**–**d3**) H&E staining of subcutaneous connective tissues in the control, MTA, iRoot SP and BC Sealer HiFlow groups at 150 days. All the groups exhibited fibrous connective tissue capsules (red arrows) with minimal inflammation. Over time, the presence of macrophages and necrotic areas generally declined. (100×, bar, 300 μm; insets show tissue details at 400×, bar, 60 μm).

Figure 3 shows the inflammatory infiltration results at each time point, and the data are compiled in Table 3. On the 7th day, compared with the control group, more visual fields had severe inflammatory scores in the MTA group, followed by the iRoot SP group, and the BC Sealer HiFlow group had the fewest visual fields with severe inflammatory scores. On the 30th day, more than 50% of the specimens in the control group and over 85% of the specimens in the MTA group exhibited inflammatory responses that ranged from absent to mild. In the iRoot SP and BC Sealer HiFlow groups, the inflammatory responses remained mild to moderate. Finally, on the 150th day, more than 90% of the specimens in all the groups showed no or mild inflammation. Moreover, bundles of long spindle-shaped fibroblasts were observed at the interface between the materials and tissue.

Macrophages were observed in all groups at all experimental time points; however, the number and score of the macrophages gradually decreased over time, which was similar to the necrotic area results. Among the groups, the MTA and BC Sealer HiFlow groups scored higher than the negative control and iRoot SP groups on day 7, while there was no significant difference between all the material treatment groups and the control group on day 30. On day 150, all the material treatment groups scored lower than the control group (*p* ˂ 0.05). As time progressed, the proportion of the necrotic area in each group gradually decreased, reaching a minimum at 150 days. In addition, the only significant differences on day 30 were those observed between all the material treatment groups and the control group. The results of the statistical analysis described above demonstrated that the local inflammatory response and the necrotic areas generated in response to all the biomaterials gradually decreased, suggested an overall trend towards no aggravation and even alleviation of the inflammatory responses elicited by these three endodontic biomaterials.

### 3.4. iRoot SP and HiFlow Promoted M2-like Macrophage Polarization

The inflammatory response elicited by the biocompatible implant model in vivo mainly involved macrophage infiltration, and this response is also known as the foreign body reaction. This response is key for determining biocompatibility and bioactivity by regulating the macrophage secretion of inflammatory factors that affects the final success of the implanted materials. Therefore, in this study, the effects of MTA, iRoot SP and BC Sealer HiFlow on macrophage polarization after subcutaneous implantation were investigated.

**Figure 4:**
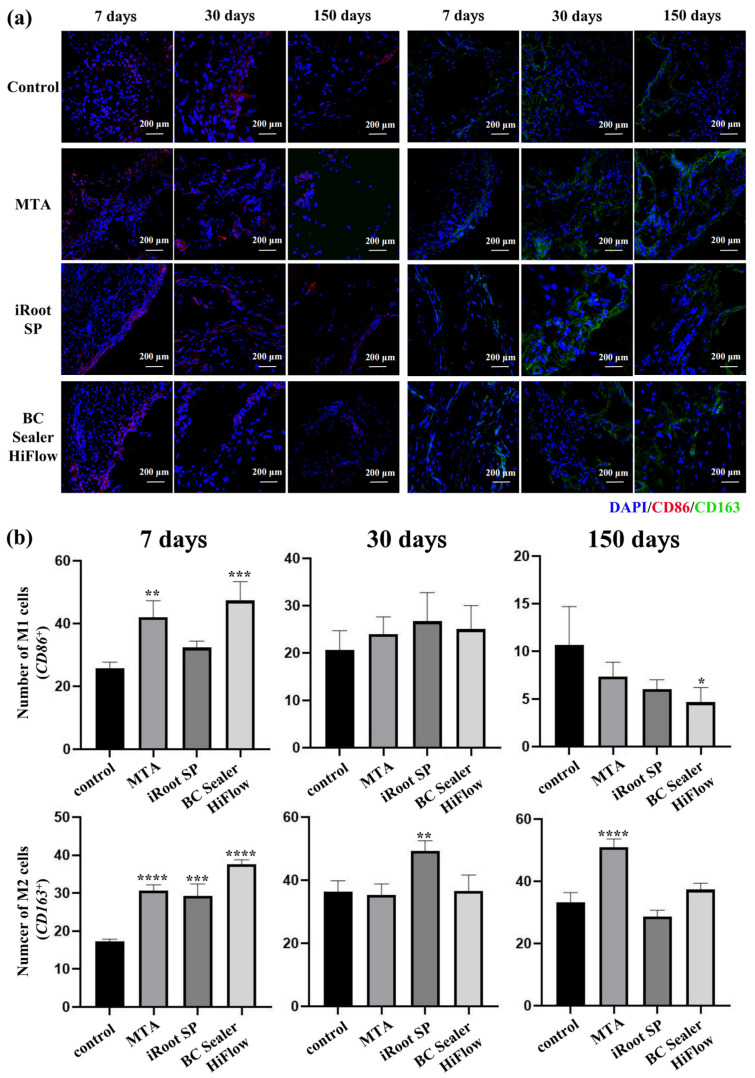
In vivo macrophage polarization in response to three biomaterials. (**a**) Localizations of CD86-positive and CD163-positive cells in rat subcutaneous connective tissues at 7, 30 and 150 days after implantation of empty dentine tubes (control), MTA, SP and HiFlow. The nuclei were stained with DAPI (400×, bar, 200 μm). (**b**) Fluorescence semiquantitative statistical analysis of M1 and M2 macrophages at 7, 30 and 150 days. * *p* ˂ 0.05, ** *p* ˂ 0.01, *** *p* ˂ 0.001, **** *p* ˂ 0.0001 versus the control group.

As shown in Figure 4, the numbers of M1 macrophages (CD86-positive cells) and M2 macrophages (CD163-positive cells) were significantly higher in the material-treated samples on the 7th day (*p* ˂ 0.05), except in the iRoot SP group, in which the number of M1 macrophages was only slightly higher than that in the control group (*p* ˃ 0.05). On the 30th day, more M2 macrophages were observed in the iRoot SP group than in the control group. At 150 days, the number of M1 macrophages was lower in all the endodontic material groups, with only the BC Sealer HiFlow group presenting a significant difference. The presence of M2 macrophages remained almost unchanged (*p* ˃ 0.05), with only the MTA group showing an increase (*p* ˂ 0.05). The above results suggest that MTA, iRoot SP and BC Sealer HiFlow may promote macrophage differentiation towards the M2 phenotype.

**Figure 5:**
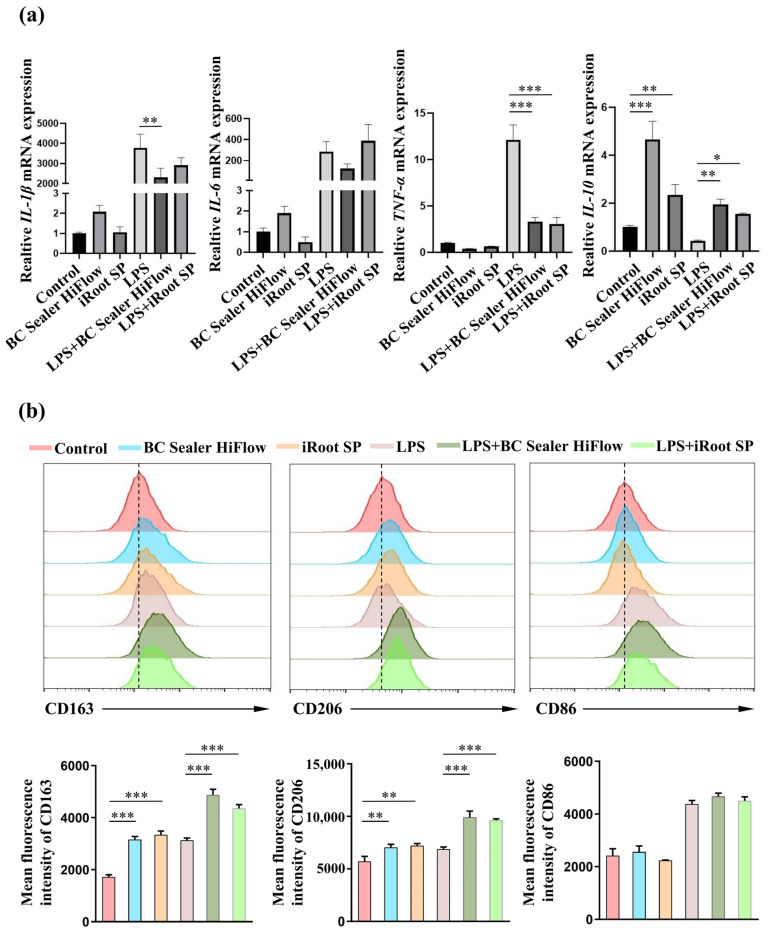
In vitro macrophage polarization in response to BC Sealer HiFlow and iRoot SP. The BMDMs were stimulated with BC Sealer HiFlow or Root SP in the presence or absence of LPS for 12 h. (**a**) The mRNA levels of *IL-1β*, *IL-6*, *TNF-α*, and *IL-10* in BMDMs were analyzed by qPCR. (**b**) The fluorescence intensity of CD163, CD206 and CD86 on the surface of BMDMs was assessed by flow cytometry. * *p* ˂ 0.05, ** *p* ˂ 0.01, *** *p* ˂ 0.001.

The ionic dissolution products are thought to be critical to the biological behavior of calcium silicate-based materials, which prompted us to explore the effects of BC Sealer HiFlow extracts on macrophage polarization in vitro. Therefore, BMDMs were isolated from mouse bone marrow, cultured, and then stimulated with iRoot SP and BC Sealer HiFlow extracts in the presence or absence of LPS. The RT–qPCR results were approximately consistent with the in vivo trends of macrophage polarization described above, as the calcium silicate-based bioceramic material BC Sealer HiFlow suppressed the mRNA expression of *IL-1β* and *TNF-α* in the presence of LPS but enhanced the level of *IL-10* both in the presence and absence of LPS after stimulation for 12 h (Figure 5a). iRoot SP inhibited the expression of *TNF-α* after LPS stimulation but promoted the expression of *IL-10* in the presence or absence of LPS. Neither bioceramic exerted an obvious effect on *IL-6* expression. Moreover, there was no significant difference between the two bioceramics in the presence of LPS. Using flow cytometry, we found that the mean fluorescence intensities of CD163 and CD206 expression in BMDMs increased after 12 h of treatment with BC Sealer HiFlow or iRoot SP regardless of whether LPS was present. However, no obvious change in CD86 expression was observed (Figure 5b). These data indicate that both iRoot SP and BC Sealer HiFlow extracts induce M2-like macrophage polarization and inhibit proinflammatory factor expression.

## 4. Discussion

With the introduction of calcium silicate-based sealers, the probability that these sealers will extrude beyond the root canal space to contact periapical tissues may increase because they are injected into the root canal [20]. Therefore, evaluations of the biocompatibility of the newly recommended root canal sealer BC Sealer HiFlow are necessary. Subcutaneous tissue implantation is one of the most reasonable methods for assessing the in vivo biocompatibility of root canal sealers, and the subcutaneous tissue–tube interface simulates the reactions that occur after root canal obturation [21,22,23]. The traditional biofriendly endodontic cement ProRoot MTA, which consists of the fine hydrophilic powders tricalcium silicate, tricalcium aluminate, and tricalcium oxide as well as other oxides, has remained the gold standard for direct pulp capping, root-end filling and apexification compared with various newer cements [24,25]. The results of studies on the cytotoxicity and bioactivity of ProRoot MTA, which was used as the control in this study, have confirmed that this material has favorable biocompatibility and bioactivity, which are attributed to the formation of hydroxyapatite or carbonated apatite [26,27]. Notably, all of the root canal repair materials or root canal sealers tested in this study contain calcium silicate.

Systemic toxicity and local toxicity are two main characteristics that determine the in vivo biocompatibility of an endodontic material. White ProRoot MTA has been histologically proven to not cause morphological changes in the liver and does not significantly alter serum GOT and GPT levels [28]. Consistent with the conclusion that white MTA is biocompatible, our results further indicate the nonsystemic toxicity of ProRoot MTA, iRoot SP and BC Sealer HiFlow, and that the focal inflammatory infiltration elicited by these materials was mild and limited. Regarding local toxicity in the subcutaneous connective tissue, inflammatory infiltration and tissue necrosis occurred in all the groups and reached a peak at day 7, with no significant difference. Our results are consistent with a previous study that examined the in vivo biocompatibility of MTA and iRoot SP, both of which resulted in a slightly higher proportion of inflammatory regions compared with empty tubes, but there was no significant difference [4]. However, BC Sealer HiFlow induced higher inflammatory responses than iRoot SP at approximately one week in a recent study [18]. The immediate implantation with sealer pastes in that study may account for the difference. The formation of an apatite layer on the surface of calcium silicate-based cements after exposure to phosphate-buffered solution or implantation under subcutaneous tissues could be completed within 1 week [29,30]. This result proves that the early precipitation of apatite crystalline structures of bioactive compounds may account for the histological results in this study. On day 30, a higher proportion of inflammatory reactions, ranging from mild to moderate, was observed in both the bioceramic groups compared with the control group, which was consistent with the higher amounts of *IL-6* observed by immunohistochemical staining in the iRoot SP group than in the control group in Silva’s experiment [14]. Chronic inflammatory reactions that ranged from absent to mild were observed in the HiFlow group at 30 days in Santos’ study, and differences in sampling areas and visual fields may result in different rating results [18]. More importantly, the inflammatory response gradually decreased over time in both experiments.

After 150 days, there was no severe inflammatory infiltration in any of the groups, and over 90% of the areas received scores ranging from absent-to-mild inflammation. Because an alkaline pH is desirable for promoting repair after endodontic treatment, the biocompatibility of bioceramics may be associated with the long-term alkaline environment created by the bioceramic materials [31]. Furthermore, when assessing material biocompatibility, later harmful effects are considered to be more important than the initial effects [32]. To the best of our knowledge, this is the first study to examine the long-term (over 90 days) in vivo biocompatibility of EndoSequence BC Sealer HiFlow. Furthermore, the fibrous connective tissues that surrounded the materials in the present study indicated that the bioceramics were well tolerated by tissues [33]. Necrosis was also present at all the experimental time points, which is understandable, owing to foreign body implantation and surgical trauma [34]. Our results revealed evidently more necrotic areas in all the endodontic material groups than in the control group on day 30. This result may have been associated with the endodontic material particles that were extruded to connective tissues, causing macrophage aggregation and suggesting that particle elimination was difficult [4]. After this time point, the necrotic areas tended to decrease and were smallest at 150 days. Therefore, the above experimental phenomena observed in the present study suggested that HiFlow has an excellent in vivo biocompatibility that is comparable to that of iRoot SP, which is partially consistent with a recent study.

To the best of our knowledge, this is the first study to evaluate EndoSequence BC Sealer HiFlow biocompatibility starting with an in vivo observation of the surface crystal morphology after subcutaneous transplantation. Interestingly, the results indicate that these materials have an ability to undergo in vivo biomineralization, which was under debate in some earlier studies [35]. Previous in vitro studies found that the formation of nanoscale structures on the surfaces of calcium phosphate- or calcium silicate-based endodontic materials immersed in simulated body fluid favors the recruitment and adhesion of mesenchymal stem cells (MSCs), human periodontal ligament cells (hPDLCs), and human dental pulp stem cells (hDPSCs) and further promotes metabolic kinetics [24,36,37]. The in vivo formation of apatite-like precipitates on the surface of ProRoot MTA and a prototype tricalcium silicate cement was also confirmed [30]. Consistent with these in vitro and in vivo findings, our results further indicate that the three biomaterials tested here displayed various capacities to form nanoapatite crystals with similar ultrastructures in vivo, and of the investigated materials, BC Sealer HiFlow had the highest density due to its higher P content. Sediments on the surfaces of bioceramic materials that are much more similar in composition to bone-like hydroxyapatite (HA) play a more effective role in biological activity [38]. Similar to other in vitro studies that have found comparable chemical compositions of the surface layers of iRoot SP and HiFlow, our in vivo study further revealed that both bioceramics have a similar elemental composition by using EDX to analyse the circular crystals formed on the surface [12,39]. HiFlow had a Ca/P ratio of 1.688, which is surprisingly close to the human bone Ca/P ratio of 1.67, and this feature appears to benefit its bioactivity and biocompatibility [38].

Macrophages were observed in all the specimens at all the time points, and the macrophage numbers in all the groups peaked on day 7 because macrophages are the main infiltrating cells that secrete inflammatory cytokines at the interface between biomaterials and tissues [17]. A previous study investigating the in vivo biocompatibility of iRoot SP and BC Sealer HiFlow suggested that the bioceramic groups had more macrophages until day 30 [18]. However, the results of our study are inconsistent with those of previous studies, as the macrophage scores in the three biomaterial treatment groups were not significantly different from those in the control group. Different implant dimensions may account for the difference in the observed trends. A slightly higher number of macrophages was found in the BC Sealer HiFlow group than in the iRoot SP group on day 30, which was similar to the result reported in a recent study and may be associated with the higher solubility of HiFlow, which can promote the release of more material components, as demonstrated in vitro by other scholars [18,39].

Macrophages are major participants in foreign body reactions, and they have the potential to produce inflammatory factors; M1 macrophages are mainly involved in the proinflammatory response, while M2 macrophages mainly promote wound healing [40,41]. More importantly, the balance between these two phenotypes is the key to tissue healing [42]. During the process of bone fracture healing, M1 macrophages first stimulate the immune response, which is conducive to early-stage healing; then, M2 macrophages secrete osteogenic-related signaling molecules or proteins, such as *BMP2* and *TGF-β*, which are very important for effective osteogenic mineralization in the later stage [42]. In our study, the highest number of M1 macrophages in all the groups was found on day 7, and then, this number decreased over time, with significantly higher numbers in the MTA and HiFlow groups initially and obviously lower numbers only in the HiFlow group on day 150. The number of M2 macrophages gradually increased until day 30 and then remained nearly consistent from day 30 to day 150, with significantly higher numbers in all the material treatment groups than in the control group at initial stages. After dorsal subcutaneous implantation in rats, the foreign body reaction was initiated by macrophages through the phagocytosis of foreign particles, amplifying the inflammatory reaction and recruiting additional immune cells [40]. As the foreign body reaction decreased, M1 macrophages transitioned towards the M2 phenotype, thereby promoting the secretion of anti-inflammatory factors and tissue regeneration [43]. Then, probably due to the release of bioactive ions, which were found in the MTA and calcium silicate bioactive ceramic extracts, the conversion of macrophages to the M2 phenotype continued [17,44]. M2 macrophages dominated in the later stage of implantation and enabled the secretion of anti-inflammatory factors, the recruitment of progenitor cells and the production of growth factors, providing convincing evidence for the conclusion that iRoot SP and HiFlow possessed good biocompatibility in this experiment. Our in vitro results subsequently verified the satisfactory anti-inflammatory effects of these two bioceramics, which were mediated by downregulating proinflammatory factor expression and upregulating the levels of the suppressive inflammatory factor *IL-10* and M2 macrophage surface markers (CD206 and CD163); these observations confirmed our earlier histological research. However, as CD86 expression was not affected, as shown by flow cytometry, and *IL-6* expression was not significantly different, as shown by RT–qPCR, we could conclude only that BC Sealer HiFlow and iRoot SP promoted M2-like macrophage polarization. Although M2-like macrophage phenotypes vary slightly, these cells have been reported by other studies of subcutaneous foreign body reactions to have the capability to relieve inflammation by playing a role comparable to that of M2 macrophages [45,46]. The further effects of M2-like macrophage polarization driven by BC Sealer HiFlow and iRoot SP, which resulted in the suppression of inflammation, may improve the bioactivity and biocompatibility of both BC Sealer HiFlow and iRoot SP in vivo. Nonetheless, further investigations are required to elucidate the underlying mechanisms. In summary, the novelty of our study lies in revealing that the favorable biocompatibility of BC Sealer HiFlow and iRoot SP may benefit from the promotion of M2-like macrophage generation. Furthermore, our study also confirmed the satisfactory ability of HiFlow to form bioactive apatite crystals in vivo and its lack of obvious toxicity under long-term subcutaneous transplantation conditions.

## 5. Conclusions

Within the limitations of failing to fully mimic the reaction between sealers and periapical tissues in the current study, it may be concluded that BC Sealer HiFlow, iRoot SP and MTA are biocompatible with subcutaneous tissues, have a satisfactory ability to form similar ultrastructural bone-like apatite layers and promote M2-like macrophage polarization in vivo. The inhibition of inflammation caused by both BC Sealer HiFlow and iRoot SP was verified by measuring the expression of related inflammatory factors and macrophage surface markers in vitro. Further investigations are required to reveal the mechanism by which these bioceramics promote the generation of M2-like macrophages.

## Figures and Tables

**Table 1:** Primer sequences for each target inflammatory factor.

Target Gene	Sequence	Product Size (bp)	GeneBank Number
*β-actin*	Forward: GTGACGTTGACATCCGTAAAGA Reverse: GCCGGACTCATCGTACTCC	245	NM_007393
*IL-1β*	Forward: GAAATGCCACCTTTTGACAGTG Reverse: TGGATGCTCTCATCAGGACAG	116	NM_008361
*IL-6*	Forward: CTGCAAGAGACTTCCATCCAG Reverse: AGTGGTATAGACAGGTCTGTTGG	131	NM_031168
*TNF-ɑ*	Forward: CTGAACTTCGGGGTGATCGG Reverse: GGCTTGTCACTCGAATTTTGAGA	122	NM_013693
*IL-10*	Forward: AGCCTTATCGAAATGATCCAGT Reverse: GGCCTTGTAGACACCTTGGT	229	NM_010548

**Table 2:** EDX analysis of MTA, iRoot SP and BC Sealer HiFlow.

MTA		iRoot SP		BC Sealer HiFlow	
Element	Atomic%	Element	Atomic%	Element	Atomic%
C-K	22.41	O-K	70.68	O-K	62.29
O-K	58.06	-	-	-	-
Si-K	0.66	Si-K	0.10	Si-K	0.10
P-K	0.64	P-K	2.85	P-K	7.25
S-K	0.23	-	-	-	-
Ca-K	18.00	Ca-K	6.24	Ca-K	12.24
Zr-L	0.00	Zr-L	20.13	Zr-L	18.12
Totals	100.00	Totals	100.00	Totals	100.00

**Table 3:** Percentage of samples in each group categorized according to the inflammatory score, macrophage score and necrosis at each time point.

Material	Inflammatory Score *				Macrophage ^†^			Necrosis ^#^	
	0	1	2	3	0	1	2	0	1
7 days									
Control	20.6	20.6	23.5	35.3	41.2 ^a^	47.1 ^a^	11.8 ^a^	61.8	38.2
MTA	8.8	8.8	35.3	47.1	17.6 ^b^	82.4 ^b^	0 ^b^	41.2	58.8
iRoot SP	5.9	11.8	41.2	41.2	17.6 ^a^	70.6 ^a^	11.8 ^a^	47.1	52.9
BC Sealer HiFlow	8.8	17.6	41.2	32.4	14.7 ^b^	85.3 ^b^	0 ^b^	50.0	50.0
30 days									
Control	29.4 ^a^	38.2 ^a^	32.4 ^a^	0 ^a^	67.6 ^a^	32.4 ^a^	0 ^a^	88.2 ^a^	11.8 ^a^
MTA	34.8 ^a^	52.2 ^a^	13.0 ^a^	0 ^a^	80.4 ^a^	19.6 ^a^	0 ^a^	60.9 ^b^	39.1 ^b^
iRoot SP	14.7 ^à^	38.2 ^à^	47.1 ^à^	0 ^à^	91.1 ^a^	8.8 ^a^	0 ^a^	55.9 ^b^	44.1 ^b^
BC Sealer HiFlow	8.8 ^à^	44.1 ^à^	47.1 ^à^	0 ^à^	58.8 ^ã^	41.2 ^ã^	0 ^ã^	61.8 ^b^	38.2 ^b^
150 days									
Control	68.9 ^a^	23.0 ^a^	8.2 ^a^	0 ^a^	68.9 ^a^	31.1 ^a^	0 ^a^	95.1	4.9
MTA	41.9 ^a^	48.8 ^a^	9.3 ^a^	0 ^a^	87.5 ^b^	12.5 ^b^	0 ^b^	95.8	4.2
iRoot SP	58.5 ^a^	37.7 ^a^	3.8 ^a^	0 ^a^	96.2 ^b^	3.8 ^b^	0 ^b^	83.0	17.0
BC Sealer HiFlow	73.7 ^à^	26.3 ^à^	0 ^à^	0 ^à^	97.4 ^b^	2.6 ^b^	0 ^b^	92.1	7.9

Different letters “a” and “b” in the same column indicate that there is a statistically significant difference between the endodontic material treatment groups versus the control group in terms of the inflammatory score, the macrophage score or the necrosis score at each time point (*p* < 0.05). “à” at 30 days indicates that there is no significant difference among MTA, SP and HiFlow versus the control, but there are significant differences between MTA and SP or HiFlow in terms of the inflammatory score. “ã” at 30 days indicates that there is no significant difference among MTA, SP and HiFlow versus the control, but there is a significant difference between HiFlow and SP in terms of the macrophage score. “à” at 150 days indicates that there is no significant difference among MTA, SP and HiFlow versus the control, but there is a significant difference between HiFlow and MTA. * Inflammatory score: 0, no reaction (no or fewer than 10 inflammatory cells); 1, mild (between 10 and 25 cells); 2, moderate (between 26 and 65 cells); 3, severe (more than 65 cells). ^†^ Macrophage score: 0, no reaction (no or fewer than 10 macrophages); 1, mild (between 10 and 30 cells); 2, moderate or severe (more than 30 cells). ^#^ Necrosis area: 0, absent; 1, present.

## Data Availability

The data presented in this study are available upon request from the corresponding author.
